# Planetary sleep medicine: Studying sleep at the individual, population, and planetary level

**DOI:** 10.3389/fpubh.2022.1005100

**Published:** 2022-10-18

**Authors:** Nicola Luigi Bragazzi, Sergio Garbarino, Luca Puce, Carlo Trompetto, Lucio Marinelli, Antonio Currà, Haitham Jahrami, Khaled Trabelsi, Bruce Mellado, Ali Asgary, Jianhong Wu, Jude Dzevela Kong

**Affiliations:** ^1^Laboratory for Industrial and Applied Mathematics (LIAM), Department of Mathematics and Statistics, York University, Toronto, ON, Canada; ^2^Department of Neuroscience, Rehabilitation, Ophthalmology, Genetics and Maternal/Child Sciences (DINOGMI), University of Genoa, Genoa, Italy; ^3^Istituto di Ricovero e Cura a Carattere Scientifico (IRCCS) Ospedale Policlinico San Martino, Genoa, Italy; ^4^Department of Medical-Surgical Sciences and Biotechnologies, Academic Neurology Unit, Ospedale A. Fiorini, Terracina, Italy; ^5^Sapienza University of Rome, Rome, Italy; ^6^Ministry of Health, Manama, Bahrain; ^7^College of Medicine and Medical Sciences, Arabian Gulf University, Manama, Bahrain; ^8^High Institute of Sport and Physical Education of Sfax, University of Sfax, Sfax, Tunisia; ^9^Research Laboratory: Education, Motricity, Sport and Health, EM2S, LR19JS01, University of Sfax, Sfax, Tunisia; ^10^School of Physics and Institute for Collider Particle Physics, University of the Witwatersrand, Johannesburg, South Africa; ^11^Subatomic Physics, iThemba Laboratory for Accelerator Based Sciences, Somerset West, South Africa; ^12^Disaster and Emergency Management Area and Advanced Disaster, Emergency and Rapid-Response Simulation (ADERSIM), School of Administrative Studies, York University, Toronto, ON, Canada

**Keywords:** one health, global health, planetary health, sleep medicine, circadian rhythms, biological timekeeping, chronomedicine

## Abstract

Circadian rhythms are a series of endogenous autonomous oscillators that are generated by the molecular circadian clock which coordinates and synchronizes internal time with the external environment in a 24-h daily cycle (that can also be shorter or longer than 24 h). Besides daily rhythms, there exist as well other biological rhythms that have different time scales, including seasonal and annual rhythms. Circadian and other biological rhythms deeply permeate human life, at any level, spanning from the molecular, subcellular, cellular, tissue, and organismal level to environmental exposures, and behavioral lifestyles. Humans are immersed in what has been called the “circadian landscape,“ with circadian rhythms being highly pervasive and ubiquitous, and affecting every ecosystem on the planet, from plants to insects, fishes, birds, mammals, and other animals. Anthropogenic behaviors have been producing a cascading and compounding series of effects, including detrimental impacts on human health. However, the effects of climate change on sleep have been relatively overlooked. In the present narrative review paper, we wanted to offer a way to re-read/re-think sleep medicine from a planetary health perspective. Climate change, through a complex series of either direct or indirect mechanisms, including (i) pollution- and poor air quality-induced oxygen saturation variability/hypoxia, (ii) changes in light conditions and increases in the nighttime, (iii) fluctuating temperatures, warmer values, and heat due to extreme weather, and (iv) psychological distress imposed by disasters (like floods, wildfires, droughts, hurricanes, and infectious outbreaks by emerging and reemerging pathogens) may contribute to inducing mismatches between internal time and external environment, and disrupting sleep, causing poor sleep quantity and quality and sleep disorders, such as insomnia, and sleep-related breathing issues, among others. Climate change will generate relevant costs and impact more vulnerable populations in underserved areas, thus widening already existing global geographic, age-, sex-, and gender-related inequalities.

## Circadian and other biological rhythms

Circadian rhythms are a series of endogenous autonomous oscillators that are generated by the molecular circadian clock which coordinates and synchronizes internal time with the external environment in a 24-h daily cycle (that can also be shorter or longer than 24 h) (1). Besides daily rhythms, there exist as well other biological rhythms that have different time scales, including seasonal and annual rhythms (2). Circadian and other biological rhythms deeply permeate human life, at any level, spanning from the molecular, subcellular, cellular, tissue, and organismal level to environmental exposures, and behavioral lifestyles (1). Humans are immersed in what has been called the “circadian landscape” (1), with circadian (and other biological) rhythms being highly pervasive and ubiquitous, and affecting every ecosystem on the planet, being phylogenetically widespread, from protists and bacteria to plants, insects, fishes, birds, mammals, and other animals (2, 3). From an ecological and evolutionary perspective, circadian and other biological rhythms are extremely ancient and are hypothesized to enhance the fitness of the organisms, enabling them to better and more efficiently use and allocate resources and allowing for proper internal temporal order (4).

To put it in other words, the universe is written in the language and alphabet of circadian and other biological rhythms. Physiological functions as well as physiopathological dysfunctions (5), disease expression and severity (6), course/evolution and prognosis (6), and drug action and effects (in terms of pharmacokinetics and pharmacodynamics) (7) exhibit variations by time of day (8).

In both humans and animals, biological rhythms are regulated and fine-tuned by the so-called “inner biological clock,” which is situated at the level of the suprachiasmatic *nuclei* (SCN) of the hypothalamus (9). This neuro-structure projects to the pineal gland (the so-called “circadian neural message”), which secretes melatonin in a sequential and pulsatile fashion (the so-called “circadian humoral message”) (10), both into the capillary bed within the gland and into the cerebrospinal fluid of the third ventricle, aided by a number of epithalamic structures, such as interpinealocyte *canaliculi* and evaginations of the posterodorsal third ventricle itself, and *via* tanycytes located in the pineal recess and a discontinuous ependymal lining (11, 12).

Besides central clocks, there exist peripheral oscillators (“peripheral circadian clocks”) (13), which include cardiometabolic, endocrine, immune, and reproductive systems (14). The precise relationship between central and peripheral oscillators has to be elucidated yet, but, briefly, two major competing models exist: the “master-slave” model (15) and Albrecht's “orchestra” model (16–18). In the former model, the circadian network is hierarchically organized and the synchronization power is entirely in the power of the master clock (the “central pacemaker”), with “subordinate peripheral oscillators” being relatively insensitive to external/environmental cues. In the latter model, the central clock behaves as the orchestra conductor, with each peripheral clock behaving as an orchestra member and being able to play its own instrument synchronizing with the conductor as well as independently.

There is a considerable degree of individual variability in circadian rhythms, which can be investigated by a series of biomarkers, including sleep-wake patterns (19), body temperature cycles (20, 21), hormonal (22) and blood pressure (23) fluctuations, and gene activation, regulation, translation, and expression patterns (24–28), and microbiota oscillations (29–31). These are the gatekeepers of biological rhythms.

The sleep-wake cycle can be measured and expressed as “circadian typology” or chronotypes, also known as morningness-eveningness, which are distributed normally on a *continuum* (32), enabling the categorization of individuals into three major groups: namely, (i) morning chronotype, (ii) evening chronotype, and (iii) neither chronotypes (or intermediate chronotype). Individuals belonging to the former chronotype are known as “larks,” being more likely to wake up in the early morning and preferring to go to sleep in the early evening, thus exhibiting a diurnal preference. Individuals belonging to the latter chronotype are known as “owls,” and tend to wake up later in the morning and go to sleep later in the evening, displaying a nocturnal preference (32, 33).

Utilizing large datasets, big data, and artificial intelligence can enable capturing the “chronobiome,” which can be defined as the collection and analysis of individual physiological traits in a 24-h rhythmic pattern to embrace the complexity and temporal dimension (time-dependence) of deep phenotyping (34) under free-ranging conditions (35). Technological advancements, including multiscale “omics” and remote sensors, offer new unprecedented opportunities to explore new knowledge domains (36).

Among the different circadian rhythms, sleep, as a light-related and regulated circadian rhythm, will be overviewed in the next section.

## Sleep

Far from being a mere, passive “state of neural inactivity,” sleep is a vital, multi-factorial, and complex series of neurophysiological events, which involve several interacting and cascading processes at the level of the central nervous system (37). High-quality, refreshing sleep is of paramount importance for daily proper functioning (37), since it regulates homeostasis, repair mechanisms, synaptic strength (38–40), and removal of potentially harmful interstitial metabolic waste products from the brain *intima* (41). According to the “synaptic homeostasis hypothesis” (SHY), sleep is the “price to pay” for ensuring brain dynamic plasticity, consolidation of learning, and readiness to learn new things the day after. Replenishing and cleansing of the brain is another essential role of sleep, which occurs through convective fluid transport (a process known as “glymphatic clearance”) (41, 42). Of note, unsurprisingly, brain glymphatic and lymphatic fluid flows are under circadian control (43).

Sleep represents approximately one-third of our life (44) and is observed as well in all species of living organisms on the planet, including reptiles, amphibians, fishes, birds, and other animals, like *Homo sapiens* (44). As for circadian and other biological rhythms, also sleep has an ancient origin (45). The master clock regulates the sleep-wake cycle, shaped by circadian rhythms and planet rotation (46). As such, sleep is influenced by circadian processes as well as by other biological rhythms, such as seasonality: sleep tends to be longer in cold seasons (winter) and shorter in warm/hot seasons (summer), potentially due to increased day length and/or increased temperature. Of note, these effects are particularly pronounced in children and in the elderly, as well as in members of preindustrial societies, or in the absence of artificial light (47).

Leveraging big data analytics can enable capturing the “sleepome,” which can be defined as “the dissection of differences and oscillations in sleep dynamics and architecture at the individual level” (48), paving the way for “precision sleep medicine” (49). This approach and related techniques can be applied also at the community and population level, even though there are very few nationwide studies, relying on large datasets. Even fewer studies have explored the link between seasonality/local weather and sleep using objective, continuous, unobtrusive measurements (47). For instance, Mattingly et al. (47) have quantitatively assessed the sleep habits of a sample of 216 individuals across the United States over four seasons. The strongest seasonal effect could be detected for sleep duration and wake time, with a more marked effect in spring. A modest effect in terms of sleep duration could be described as related to day length (with less sleep correlating with longer days, between the winter and summer solstice). Increased outdoor temperature was found to be associated with later bedtimes and wake times.

## Circadian disruptions and sleep disturbances

Master and peripheral clock disruption, circadian misalignment/desynchrony, and disturbances of sleep-wake homeostasis can result in severe cardio-metabolic, immunological, neurological, neuro-immunological dysfunctions, and neurodegenerative diseases, increasing as well cardiovascular risk factors (1).

Sleep-related issues generate a relevant burden–both epidemiological and clinical–(50) in the nowadays post-modern, around-the-clock, 24/7 society (51) characterized by excessive working hours, rotating night shift working (52), and social jet lag (53). Besides increased workload, technological innovations and advancements, including the advent of electricity, light emitting diode (LED) technology, and artificial light at night (54), as well as societal changes, including industrial and post-industrial revolutions (55–58) have considerably compressed and shortened the length of sleep. Also, the way of sleeping has considerably changed, shifting from “segmented sleep” (known also as divided sleep or biphasic/polyphasic sleep) to “consolidated sleep” (sleeping in a consolidated block) (55–58).

All this has resulted in chronic sleep deprivation and in the insurgence of a relevant burden in terms of morbidity (cardiovascular disorders, obesity, and metabolic syndrome, among others) (59).

The effect of the new information and communication technologies (ICTs) on the sleep schedule (and, in particular, the sleep-wake cycle) is even more marked. Light emitted from screens of smartphones and mobile devices (including tablet computers) is, indeed, believed to disrupt the users' circadian rhythm, with some recent investigations showing that the usage of social networking sites may also impact social cognition, reflective functioning, and job performance, among others (60–62). Underlying biological mechanisms include the emission of blue light from cell phone screens, which restrains the production of melatonin (63).

The increasingly widespread availability of ICTs and their ubiquitous and pervasive nature have led to rising rates of nomophobia (anxiety and psychological distress related to “no mobile phone phobia”) (64), internet addiction (65), and other digital addictive behaviors, including internet-based addictive behaviors and addictive use of social media and social networks (66), which have been found to be associated with sleep issues, including insomnia (67–69).

All this has significant economic implications in terms of increased related (either direct or indirect) costs (70), which is even more relevant considering that the rate of disturbances of the circadian system, including circadian disruptions, misalignment and desynchrony, social jetlag, and chronodisruption, has been steadily increasing in the last years (71). For instance, a recent systematic review and meta-analysis pooling together 1.1 million people (individual participant data analysis from 200,358 people from 36 studies from the Netherlands, 471,759 people from the United Kingdom, and 409,617 people from the United States) (72) found that one in four people slept fewer hours than recommended. Adults reported more poor sleep quality and insomnia than short sleep duration; sleep issues did not exhibit geographic differences, with the exception of insomnia, being more commonly reported in the United States. In terms of subjective measurements (self-reported sleep times, quality, and efficiency), women reported lower values than men, which was disconfirmed by objective measurements (actigraphy), which showed opposite trends. Another interesting finding from the study is that it was able to document a progressive decline in sleep duration.

## Climate change and its drivers

Global climate change is a change in climate that cannot be explained by taking into account natural climate variability. Anthropogenic factors, including encroachment and the burning of coal and oil, on the contrary, explain a large amount of global climate change, from rising sea levels to snow melting and other profound changes affecting weather patterns. Human behaviors have gradually warmed the earth's temperature and increased the levels of atmospheric heat-trapping gases, affecting every ecosystem, fragmenting natural habitats, impacting freshwater supplies, and exerting detrimental effects on animal and human health and well-being.

In the last decades, profound societal phenomena, including gentrification and rapid, wild urbanization together with population explosion and demographic changes, have deeply modified our lifestyles (73). Approximately more than half of humanity dwells in urban areas and settlements, with this figure being expected to significantly increase up to two-thirds of the global population by 2050. For instance, in North America, at the beginning of the 20^th^ century, less than half of its population lived in urban areas, this percentage had doubled at the beginning of the new century and it can be anticipated that it will further rise in the coming decades. As beautifully stated by Teresa Coady, “the history of human settlement is the history of climate change” (74, 75). As such, to “make cities and human settlements inclusive, safe, resilient and sustainable,“ as the eleventh “Sustainable Development Goal” (SDG-11) of the United Nations (UN) ambitiously declares and has been reaffirmed during the 2016 UN “Habitat III” Conference on Housing and Sustainable Urban Development in Ecuador (the so-called “New Urban Agenda”), is becoming more and more imperative (76, 77).

## Planetary health

Human health and well-being depend on the well-being of the planet including both living and nonliving systems. In other words, planetary health can be defined as “the health of human civilization and the natural systems on which it depends” (78, 79). According to the Rockefeller Foundation-Lancet commission, planetary health is “the achievement of the highest attainable standard of health, well-being, and equity worldwide through judicious attention to the human systems—political, economic, and social—that shape the future of humanity and the Earth's natural systems that define the safe environmental limits within which humanity can flourish” (78, 79).

Planetary health can be conceived as the application of the “one health” concept on a global scale, in which there is a strong interconnection between humans, animals, and the surrounding environment.

## The impact of climate change on sleep: The effects of the rise in temperature

Anthropogenic behaviors, including urbanization and expansion of heat islands, have been producing a cascading and compounding series of effects, including detrimental impacts on human health, including mental health and cognitive functioning (80, 81). However, the role of climate change on sleep and sleeping habits has not been yet comprehensively explored. In the present review paper, we wanted to offer a way to re-read/re-think sleep medicine from a planetary health perspective.

Climate change represents a major global stressor impacting the fitness of living beings and organisms, partially due to the interference of the adaptive associations between endogenous (either central or peripheral) clocks and temperature. Endogenous temperature represents, indeed, an important *Zeitgeber* (a biological timekeeping cue), which regulates human sleep (82). There is a strong coupling between endogenous temperature and sleep, as hypothesized by the “energy allocation function of sleep” theory (83), according to which energy expenditure is finely regulated and generally conserved. Moreover, there is a correlation between the maximal rate of core body cooling, duration of wakefulness, and sleep onset, with sleep propensity peaking closely to the minimum of the core body temperature rhythm (84, 85). Climate change is relevant to sleep medicine from a planetary health perspective in that environmental (outdoor ambient) temperature impacts endogenous (body) temperature. In particular, it is during sleep that humans become more sensitive to fluctuations of the environmental (outdoor ambient) temperature, especially if the values range outside of the “thermoneutral zone” (84, 85). Hypothalamic regions, in particular, the preoptic area, act as thermoregulatory centers receiving temperature-related inputs from the core body *via* hypothalamic receptors and from the environment *via* skin receptors. The human body's thermoregulatory system is sophisticated enough to regulate and fine-tune thermogenic and heat-dissipative processes in tissues, from vasculatures to muscles, brown adipose tissue, and skin, keeping endogenous temperature relatively constant, fluctuating around a set-point, to counterbalance environmental temperature deviations (84, 85).

Obradovich et al. (86) have utilized data from 765,000 U.S. survey respondents from 2002 to 2011, and coupled them with nighttime temperature data, finding a correlation between increases in nighttime temperatures and the number of self-reported nights of insufficient sleep. This association was more marked during summer time and lower socio-economic status respondents and the elderly were disproportionately impacted.

Näyhä et al. (87) assessed the rate of heat-related complaints and symptoms in the Finnish population, sampling four thousand and seven men and women aged 25–74 years from the National FINRISK 2007 study. The ranges of thermal comfort declined with age. About 80% of the respondents complained of heat strain in warm weather, with sleep disturbances being reported in 32% of cases (the prevalence increased with age and was higher among women).

Whilst the previous studies have utilized self-reported measurements (86, 87), Milnor et al. (88) have linked billions of repeated sleep measurements from sleep-tracking wristbands consisting of more than 7 million sleep records from 68 locations to local daily meteorological data. After adjusting for confounding factors at the individual, seasonal, and time-varying level, the rise in temperature values, which is particularly relevant at night, erodes sleep duration, delaying its onset, and resulting in insufficient sleep. Limited evidence of human sleep adaptation to hotter temperatures could be found. Sex- and gender-specific effects could be described, with females being more impacted than males. Populations from low- and middle-income countries (LMICs) were also affected, along with the elderly. According to a forecasting analysis, by 2,099, climate change is anticipated to contribute to 50–58 h of sleep loss *per* person-year.

## The impact of climate change on sleep: The effects of environmental pollution

Climate change has been related to a wide array of major forms of environmental pollution, including soil and radioactive contamination, air, water, noise, light pollution, and thermal pollution, among others.

Concerning air pollution, according to a recently published systematic review of the literature (89), synthesizing 22 articles from 2010 to 2019, across the life course (from early childhood to adulthood and elderly) and a wide span of populations and locations (from North America to Europe and Asia), a positive association between environmental exposures, including exposure to ambient and indoor pollutants, such as particulate matters (PM) and gaseous components (like nitrogen dioxide, NO_2_, and ozone, O_3_), and poor sleep quality was reported. The youth was disproportionately impacted, with increased respiratory sleep issues and adverse sleep-related outcomes, whereas adults exposed to air pollutants were more likely to report sleep-disordered breathing issues. Mechanisms underlying can be disparate affecting the central ventilator centers to other neuroanatomic functional structures, as well as the upper airways (90). Several sleep disturbances have been associated with pollution, including chronic sleep deprivation (91) and obstructive sleep apnoea (OSA) syndrome (92), among others.

## The impact of climate change on sleep: The effects of emerging and re-emerging infectious diseases

Climate change, increased temperatures, and natural disasters are closely (either directly or indirectly) connected with emerging and re-emerging infectious diseases (ERIDs) (93), such as the still ongoing “Coronavirus Disease 2019” (COVID-19). In the case of vector-borne infectious diseases (like malaria, dengue, and viral encephalitides), climate change can, indeed, directly impact disease transmission dynamics by shifting the vector's geographic range, enhancing reproductive and biting rates, and shortening the pathogen incubation period (92). Climate change can have also indirect impacts, by displacing entire populations, causing massive human migration, damaging health infrastructures, putting strain on the delivery of healthcare provisions, and on agriculture as well, generating food insecurity, favoring malnutrition, and increasing human susceptibility to infectious agents (94). Increased susceptibility to infections could also occur *via* ultraviolet radiation-induced immune dysregulation (94).

In the aftermath of natural hazards and disasters, such as floods, due to potential sewage contamination of floodwaters, there is a high risk of infections caused by agents such as *Escherichia coli, Campylobacter, Amoeba, Cryptosporidium*, and *Giardia lamblia*. Vector-borne (especially mosquito-borne) infections can develop as well (95).

According to a recently published systematic review and meta-analysis (96), synthesizing two hundred and fifty studies, totaling 493,475 participants from 49 countries, the COVID-19 infectious outbreak posed a dramatically high burden of sleep disturbances, with a pooled rate computed at 40.49%, disproportionately affecting people with COVID-19, the elderly and frail, vulnerable individuals, children and adolescents, healthcare workers, and special populations with healthcare needs. The impact was particularly relevant during the COVID-19-induced restrictions, compared with the periods when the strictures were lifted. Sleep disturbances and circadian disruptions have been described also in previous infectious outbreaks, including influenza, poliomyelitis, Ebola, Zika, Nipah, and other coronaviruses (97).

Finally, it should be noted that sleep exerts an immune-supportive function, in that it has been found to promote host defense against infections as well as inflammatory insults. Sleep regulates and fine-tunes both innate and adaptive immunity (98, 99). Sleep issues, and in particular, chronic sleep deprivation, have been related to impairments of both innate and adaptive immune parameters. This can potentially result in a chronic inflammatory state and significantly increase the risk of contracting a wide range of pathologies, such as cardiometabolic, oncological, neurodegenerative, and autoimmune/autoinflammatory diseases (99). Moreover, this chronic inflammatory state increases, in turn, the susceptibility to other infections (99, 100).

## Compounding and cascading effects of global and planetary sleep medicine

Shifts in global food systems (with increasing exposure to high-energy processed food and drinks) and low physical activity have resulted in substantial rises in the obesity rate (101). Some scholars have observed that the “trend for shorter sleep duration has developed over the same time period as the dramatic increase in the prevalence of obesity” (102). There is a close relationship between the “obesity pandemic” and sleep disturbances, with overweight and obese individuals being at higher risk for sleep issues and individuals with sleep disturbances being at higher risk for developing metabolic impairments, as well (101, 102). Overweight and obese subjects can present defective diet-induced heat regulation and thermogenesis (103), and they may be, as such, more susceptible to deviations in normal temperature values caused by climate change and other planetary emergencies. Less or altered adaptive thermogenesis can lead to less physical activity, and to the production of a higher carbon footprint (104).

Climate change, in turn, is linked to rising rates of obesity and other metabolic impairments (104). Climate change increases, indeed, food insecurity and results in the adoption of unhealthy nutrition lifestyles and malnutrition (104, 105). For instance, according to the “Global Burden of Disease” (GBD) initiative, approximately one-fifth of the global burden of type 2 diabetes was attributable to air pollution, and more specifically PM_2.5_ exposure (106).

## Further developments stemming from planetary sleep medicine: Precision and digital planetary sleep medicine

If looking at sleep issues from a planetary health perspective can help advance the field, on the other hand, interventions need to be targeted, informed, and driven by locally relevant data–we call this “precision planetary sleep medicine.”

Moreover, the utilization of digital health devices and the “Internet of Things” (IoT), such as smartphone apps and smart home thermostats, could be leveraged to pave the way for a “digital planetary sleep medicine” (107). However, IoT may work only in middle- and high-income countries. Low-income countries may not have the infrastructure for this technology (108). As such, alternate ways of reaching inhabitants here are needed.

## *In vivo* planetary sleep medicine: Current challenges and future prospects

So far, evidence of the impact of climate change on human health and, more specifically, on sleep issues and circadian disturbances has been collected and assessed from in-laboratory investigations and ecological studies, where *in vivo* studies are lacking. There is, as such, the need to develop protocols for the realistic and feasible implementation of high-quality studies (109).

Moreover, a closer collaboration across fields and disciplines, involving physicians (and, in particular, neurologists, neurophysiologists, sleep specialists, neuropsychologists, and neuroscientists), biologists, climatologists, ecologists, and social and behavioral scientists, as well as other scholars, is warranted. Community and policymaker engagement is fundamental to ensure that necessary steps are undertaken in order to more precisely quantify the global burden of sleep issues attributable to climate change, and counteract or mitigate against the impacts generated by climate change (110). These interventions should be multi-component, holistic, integrated, and shaped according to a gender-, equity-, inclusion, and diversity- (GEID)-sensitive lens.

## Potential implications of planetary sleep medicine and practical recommendations

The increased global burden of circadian disruptions and sleep disturbances may significantly impact individual, and population physical and mental health and well-being. Chronic sleep deprivation, daytime fatigue, and excessive sleepiness can lead to a higher risk for workplace accidents, injuries, and even fatalities. Sleep deprivation can affect cognitive processing and reaction time, as well as job performance, productivity, task management, and meeting goals. In several industrial sectors, poor sleep quality and decreased alertness are not only health concerns, but also safety issues, impacting pilots, truck drivers, shift workers, medical residents, and other healthcare workers (111).

As such, scientists worldwide call for immediately starting to reduce and eliminate reliance on fossil fuels, transitioning to clean energy resources like renewable energies (including wind and solar energy, as well as tidal energy, hydroelectric energy, and, more in general, hydropower, geothermal energy, bioenergy, and biofuels). This is the only way we can significantly reduce carbon emissions, which represent the main cause of climate change. Besides clean energy resources, other paths to sustainability and the achievement of carbon neutrality and net-zero greenhouse gas (GHG) emissions include (i) shift toward electrification of transportation and buildings and energy efficiency improvements (including the so-called “decoupling,” to avoid inefficient utility spending), (ii) energy conservation measures, (iii) economic-financial measures such as carbon taxes, (iv) more equitable balancing of human well-being and *per capita* energy use and allocation, (v) cap and trade systems, (vi) the “carbon capture, utilization, and sequestration/storage” (CCUS) emissions reduction technology that can be applied across the energy system, by efficiently capturing carbon dioxide, utilizing it as a resource to create valuable products, and storing it, and (vii) nuclear power development and deployment (112).

Planetary crises, like the still ongoing COVID-19 pandemic and the war between Ukraine and Russia, have shown the importance of these achievements and how individuals, communities/populations, and the entire ecosystem are interconnected at the planetary level. COVID-19 has, indeed, dramatically and profoundly impacted the mechanisms underlying secure and stable energy supply chains (namely, procurement, generation, transmission, distribution, and demand), slowing progress toward universal energy access, with the war adding further setbacks.

Moreover, we must recommend educational campaigns for health care providers, decision- and policy-makers as well as the general public in many middle- and low-income countries, educating about the importance of sleep and sleep hygiene. Sleep is often ignored in these regions of the world and sleep medicine is thought to be a luxury of the rich, whilst sleep is instrumental in ensuring human life.

## Conclusions

In conclusion, the history of sleep and biological rhythms is the history of mankind and of the planet. Sleep (or sleep-like states), circadian (sleep-wake cycle), and other biological rhythms, have ancient phylogenetic roots, deeply permeating and orchestrating virtually all the ecosystems of the planet. Anthropogenic behaviors have been interfering with such biological events, undermining planetary health. Climate change, through a complex series of either direct or indirect mechanisms, including (i) pollution- and poor air quality-induced oxygen saturation variability/hypoxia (113), (ii) changes in light conditions and increases in the nighttime, (iii) fluctuating temperatures, warmer values, and heat due to extreme weather (114), and (iv) psychological distress imposed by disasters (like floods, wildfires, droughts, hurricanes, and infectious outbreaks/ERIDs–such as water, food-, vector-borne diseases and zoonotic spillovers) (94), may contribute to inducing mismatches between internal time and external environment, and disrupting sleep, causing insomnia, and sleep-related breathing issues. Climate change is expected to pose a marked burden in terms of circadian impairments and sleep disturbances, generating relevant costs (115), and impacting more vulnerable populations from underserved areas and territories, thus, widening already existing global geographic inequalities, and age-, sex- and gender-related disparities (116–118).

## Author contributions

NB and SG conceived the manuscript. LP, CT, LM, AC, HJ, KT, BM, AA, JW, and JK drafted and revised the manuscript. All authors contributed to the article and approved the submitted version.

## Funding

This research was funded by Canada's International Development Research Centre (IDRC) and the Swedish International Development Cooperation Agency (SIDA) (Grant No. 109559-001). NB and JK acknowledge support from IDRC (Grant No. 109981). JK acknowledges support from New Frontier in Research Fund-Exploratory (Grant No. NFRFE-2021-00879) and NSERC Discovery Grant (Grant No. RGPIN-2022-04559).

## Conflict of interest

The authors declare that the research was conducted in the absence of any commercial or financial relationships that could be construed as a potential conflict of interest.

## Publisher's note

All claims expressed in this article are solely those of the authors and do not necessarily represent those of their affiliated organizations, or those of the publisher, the editors and the reviewers. Any product that may be evaluated in this article, or claim that may be made by its manufacturer, is not guaranteed or endorsed by the publisher.
